# Dual-Purpose Photometric-Conductivity Detector for Simultaneous and Sequential Measurements in Flow Analysis

**DOI:** 10.3390/molecules25102284

**Published:** 2020-05-13

**Authors:** Thitirat Mantim, Korbua Chaisiwamongkhol, Kanchana Uraisin, Peter C. Hauser, Prapin Wilairat, Duangjai Nacapricha

**Affiliations:** 1 Flow Innovation-Research for Science and Technology Laboratories (FIRST Labs), Bangkok 10400, Thailand; korbua.cha@mfu.ac.th (K.C.); kanchana.ura@mahidol.ac.th (K.U.); prapin.wil@mahidol.ac.th (P.W.); 2 Department of Chemistry, Faculty of Science, Srinakharinwirot University, Sukhumwit 23 Road, Bangkok 10110, Thailand; 3 Center of Excellence for Innovation in Chemistry and Department of Chemistry, Faculty of Science, Mahidol University, Rama 6 Road, Bangkok 10400, Thailand; 4 School of Science, Mae Fah Luang University, Chiang Rai 57100, Thailand; 5 Center of Chemical Innovation for Sustainability (CIS), Mae Fah Luang University, Chiang Rai 57100, Thailand; 6 Department of Chemistry, University of Basel, Klingelbergstrasse 80, 4056 Basel, Switzerland; peter.hauser@unibas.ch; 7 National Doping Control Centre, Mahidol University, Rama 6 Road, Bangkok 10400, Thailand

**Keywords:** paired emitter–detector diode detector, contactless conductivity detector, flow-based analysis, simultaneous detection, sequential detection

## Abstract

This work presents a new dual-purpose detector for photometric and conductivity measurements in flow-based analysis. The photometric detector is a paired emitter–detector diode (PEDD) device, whilst the conductivity detection employs a capacitively coupled contactless conductivity detector (C4D). The flow-through detection cell is a rectangular acrylic block (ca. 2 × 2 × 1.5 cm) with cylindrical channels in Z-configuration. For the PEDD detector, the LED light source and detector are installed inside the acrylic block. The two electrodes of the C4D are silver conducting ink painted on the PEEK inlet and outlet tubing of the Z-flow cell. The dual-purpose detector is coupled with a sequential injection analysis (SIA) system for simultaneous detection of the absorbance of the orange dye and conductivity of the dissolved oral rehydration salt powder. The detector was also used for sequential measurements of creatinine and the conductivity of human urine samples. The creatinine analysis is based on colorimetric detection of the Jaffé reaction using the PEDD detector, and the conductivity of the urine, as measured by the C4D detector, is expressed in millisiemens (mS cm^−1^).

## 1. Introduction

Flow injection analysis (FIA) [1,2] and its later generation techniques, including sequential injection analysis (SIA) [3], lab-on-valve (LOV) [4], lab-at-valve (LAV) [5], all injection analysis (AIA) [6], simultaneous injection effective mixing flow analysis (SIEMA) [7,8] and cross injection analysis (CIA) [9,10], are effective techniques that have been used as tools for liquid handling in automated analysis. Sample introduction, reagent and sample mixing, detection and rinsing of the flow-path in every cycle are operated by computer technology. Thus, sample throughputs are up to 60 samples per hour [1,11,12,13]. Almost all kinds of detection can be coupled to the above flow-based techniques, including UV–Vis absorbance [14,15,16,17,18,19,20,21,22], fluorescence [22,23,24], chemiluminescence [25,26], electrochemical detections (amperometry) [27,28,29,30], conductivity [31] and contactless conductivity [32,33,34,35,36,37].

Some flow-based systems are equipped with two or more different types of detector in the same system, depending upon the purpose of analysis [38]. Detectors are arranged either in series [39,40,41,42,43] or in parallel [21,34,37,44]. The aim for integrating more than one detector is for multi-component analysis. For the “in-series” configuration, detectors are located along the same flow-path or in the same flow cell to accomplish sequential detections. An example of the “in-series detection” is multi-detection of different ionic species using several ion-selective electrodes but with a single reference electrode [40]. Another example of the “in-series” detection is a flow-based system that consists of an optical detection (UV–Vis absorbance) together with an amperometric detection for the analysis of total polyphenol content and antioxidant activity, respectively [39].

Unlike the “in-series” configuration, detectors with “in-parallel” configuration are placed in different flow paths. Usually, the sample is injected from the same port and the zone is split into fractions. Each of the zone-fractions is then transported into separate flowing streams for reaction (if needed), mixing (if needed) and detection. An example of the “in-parallel” configuration is the simultaneous determination of urea and creatinine in human urine using an FIA system equipped with a contactless conductivity detector (C4D) and a colorimeter [21]. Salinity, carbonate and ammoniacal nitrogen in water samples were simultaneously measured by FIA using a dual C4D detection cell arranged in an “in-parallel” configuration [34]. Salinity and carbonate were sequentially detected in C4D channel 1, and ammonical ammonia was detected after gas diffusion in the second C4D. Liquid handling by an automated SIA system is also suitable for “in-parallel” detection. Parallel analyses of sugar, colour and dissolved CO_2_ contents in soft drinks can be carried out by connecting a near infrared LED photometer and a contactless conductivity detector using an “in- parallel” configuration to completely automate flow control by SIA [44].

In this work we developed a flow cell that has dual detection capability by combining optical detection with a contactless conductivity detection in a single flow cell. The photometric detection employs paired emitter–detector diodes (PEDD), as reported by Tymecki et al. [45]. LEDs are utilized as both light sources and light detectors. The voltage of the detector LED varies with the logarithm of the intensity of the incident light. The measured voltage therefore decreases linearly with the absorbance of the solution. The liquid flow cell was fabricated from an acrylic block with a Z-shape fluidic channel. The PEEK (poly ether ether ketone) tubing attached at the inlet and outlet of the Z-shape channel is painted with silver conducting paint to form two electrode bands on the exterior wall of the tube. These two silver bands are the electrodes of the contactless conductivity detector [46,47]. An alternating voltage is applied to the electrode at the input end of the flow cell. When the channel between the two silver bands is filled with a conducting aqueous solution an AC current is generated. This current is monitored at the outlet electrode band. The amplitude of the alternating current monitored corresponds to the sample conductivity for fixed applied AC voltage and frequency. By this arrangement, it is possible to simultaneously measure the absorbance and conductivity of a solution in the flow cell.

To the best of our knowledge, this work presents the first flow cell that provides dual photometric-conductivity detection for an FIA-based system. The dual detection approach is different from the “in-series” and “in-parallel” configurations. In dual detection the detectors are combined within the same flow cell (2-in-1). C4D has previously been combined with optical detection methods, such as with UV–Vis [48,49], fluorescence [50,51,52] or UV/VIS/fluorescence [53]. However, these dual detections with C4D were all developed for and applied to capillary electrophoresis (CE) [48,49,50,53] or to microchip CE [51,52]. C4D has also been combined with amperometric detection to constitute dual detection for CE [54]. However, PEDD has never been employed in combination with C4D for dual detection in either CE or FIA-based analysis. This work presents a combination of PEDD and C4D for a simple and low-cost flow-through cell for simultaneous photometric-conductometric detection. The dual-purpose detection cell was employed with a SIA system. The first application was the simultaneous analysis of conductivity of dissolved oral rehydration salts (ORS) and the absorbance of the orange dye in the product. These data are useful for checking the quantity of electrolytes and colorant in samples of a manufacturer for consumer protection. The same flow cell was also employed for the measurements of conductivity and determination of creatinine in human urine. Measurement of urine conductivity is a fast and convenient way to ascertain the hydration status of an athlete. Urine creatinine concentration is one of the commonly required parameters for medical diagnosis. Additionally, the amount of creatinine in urine is used to correct for variation in the volume of spot urine collected for measurements of excreted drugs.

## 2. Results and Discussion

### 2.1. Design of the Detector

**Figure 1:**
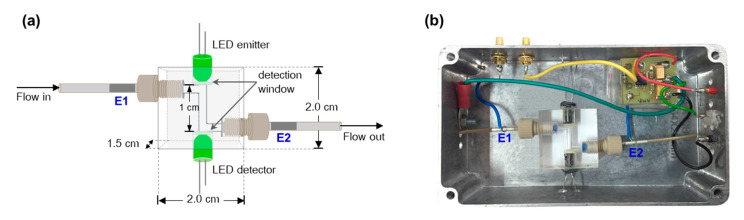
(**a**) Schematics of C4D-PEDD flow-through detection cell for the “2-in-1” detector for simultaneous detection. (**b**) Photograph of the “2-in-1” C4D-PEDD flow-through detection cell and the pre-amplifier board.

**Figure 2:**
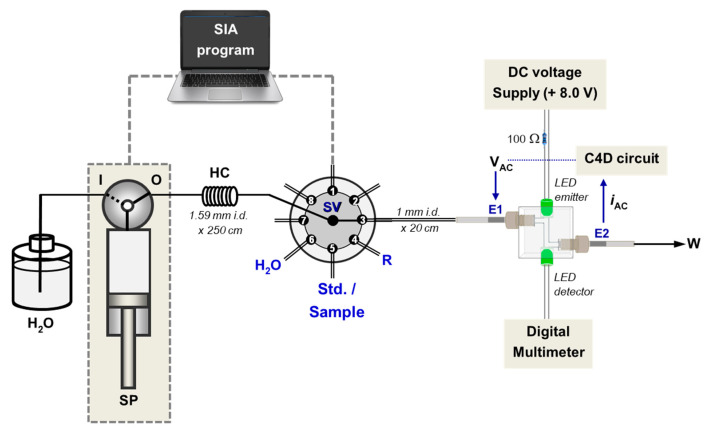
Schematic diagram of the SIA system coupled with the “2-in-1” flow cell for dual C4D-PEDD detection. Note: For the analysis of oral rehydration salt (ORS) samples, port 4 (labelled R) is not used. Port 4 is used as the inlet for the alkaline picrate reagent (R) in the sequential measurements of creatinine and conductivity of urine samples. SV—switching valve, SP—syringe pump, HC—holding coil, W—waste.

**Figure 3:**
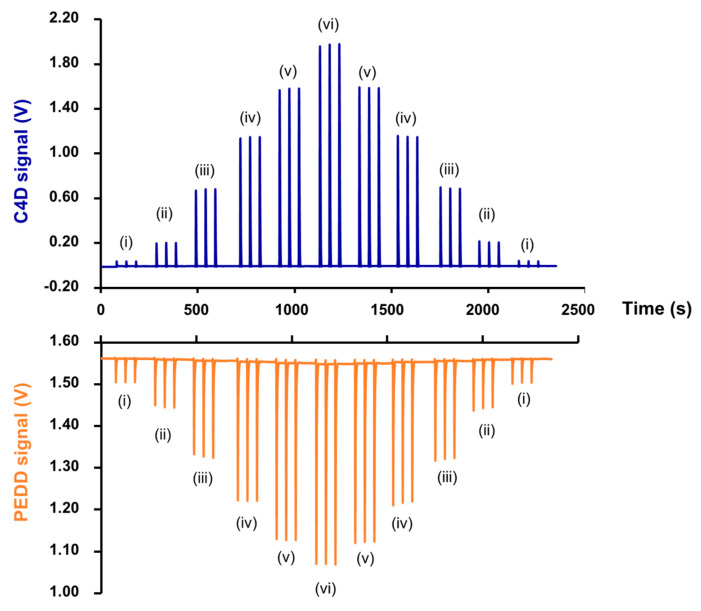
The series of signals recorded simultaneously by the C4D (top) and PEDD (bottom) detectors for triplicate injections of aqueous standard solutions in increasing order of concentrations and then in decreasing order, as carried out using SIA system (see Figure 2). The concentration of NaCl and the absorbance at 525 nm of orange dye in each standard solution: (i) 5 mM and 0.1124, (ii) 10 mM and 0.2228, (iii) 20 mM and 0.4466, (iv) 30 mM and 0.6657, (v) 40 mM and 0.8844 and (vi) 50 mM and 1.0332.

The dual-purpose detector was designed in a “2-in-1” arrangement (see Figure 1a). The electrodes E1 and E2 of the C4D are positioned at the inlet and at the outlet of the flow cell, respectively. The PEDD is used as the photometric detector for monitoring absorbance of the solution and the C4D detects the change in conductivity of the same solution passing through the flow cell. The detector is connected to a SIA system as shown in Figure 2. Upon injecting a sample solution of NaCl and orange food colorant into the SIA system, the signals of both the C4D and PEDD appear at the same time (see Figure 3). Figure 1b shows the photograph of the dual-purpose detector used throughout the work.

### 2.2. Optimization of Frequency and Voltage for the C4D

Since the configuration of the flow cell for PEDD is sufficient for this work using matching LEDs and path length of 10 mm, optimization experiments for the dual C4D-PEDD flow cell focused on the parameters affecting C4D detection. The frequency of a sinusoidal AC voltage applied to electrode E1 was varied from 100 to 500 kHz. As expected, raising the frequency from 100 to 500 kHz increased the sensitivity (slope of calibration line) of the C4D detection. At a constant voltage of 2 V_pp_ (V_pp_: peak-to-peak voltage), it was observed that while increasing the frequency from 100 to 300 kHz, the linearity range widened from 1–25 mM to 1–50 mM NaCl, respectively. The linearity ranges for 400 and 500 kHz were narrower than for 300 kHz (10–50 mM NaCl compared to 1–50 mM NaCl). However, we chose the frequency of 500 kHz, since it provided the highest sensitivity. The input voltage was then changed to 10 V_pp_. It was observed that using this condition (500 kHz and 10 V_pp_), a linear range was obtained that was suitable for the samples, and it had sufficient sensitivity. The calibration is linear with coefficient of determination (*r*^2^) of 0.999. Thus, for this study, the frequency and voltage at 500 kHz and 10 V_pp._ were employed.

### 2.3. Quantification of Salts and Dye in ORS Samples

As shown in Figure 2, the dual detection cell is connected to a sequential injection analysis (SIA) system for simultaneous measurements of conductivity and absorbance at 525 nm. The sample/standard solution (100 μL) is aspirated into the holding coil (HC) and then propelled to the flow cell by the water carrier. There is simultaneous recording of the C4D and PEDD signals (see Figure 3).

Calibration was performed by triplicate injections of a series of six aqueous solutions of NaCl and pure orange dye. The concentrations of NaCl and the colored dye were first increased from the lowest to highest concentrations (see details of the concentrations in the caption of Figure 3) and then in decreasing order. Figure 3 shows the peak profiles for the 33 consecutive injections. It should be noted that the C4D signal increases with increasing concentration of NaCl solution, whereas the PEDD signal decreases with increasing absorbance of the dye (as described in [45]). The profiles did not show any signs of carryover for both C4D and PEDD detectors, when the order of injections was from high to low concentrations, respectively.

In order to convert the measured C4D signal into conductivity unit, i.e., mS cm^−1^, the conductivity of a set of saline solutions (with concentrations covering the range employed in the C4D calibration) was measured using a commercial conductivity probe. The calibrating equation is y(mS cm^−1^) = (0.094 ± 0.006)x + (0.18 ± 0.06), *r*^2^ = 0.9996, using 10.0–50.0 mM standard NaCl solutions. From this equation the C4D signal can be converted to conductivity unit, mS cm^−1^. The plot of the C4D signal (V) against conductivity is shown in Figure S1a. The linear calibration equation is y(V) = (0.47 ± 0.01)x − (0.27 ± 0.02), *r*^2^ = 0.9993. The slopes and intercepts of the calibration lines obtained with the data for increasing concentrations and the data for decreasing concentrations are not statistically different, as shown in Figure S2a,b, respectively.

The linear calibration of the PEDD is the plot of the negative of the difference (−ΔV) of the signal minimum (in volts, V) from the baseline value (see Figure 3) against the absorbance (au, at 525 nm) of the standard solutions of the orange dye (measured previously with a spectrophotometer). A typical calibration equation is: −ΔV = ((4.86 ± 0.12) × 10^−1^)⋅au + ((0.92 ± 0.66) × 10^−2^), *r*^2^ = 0.998. Similarly to the C4D measurements, the slopes and intercepts of the calibration lines obtained with the data for increasing concentrations and that for decreasing concentrations are not statistically different, as shown in Figure S2c,d, respectively.

**Figure 4:**
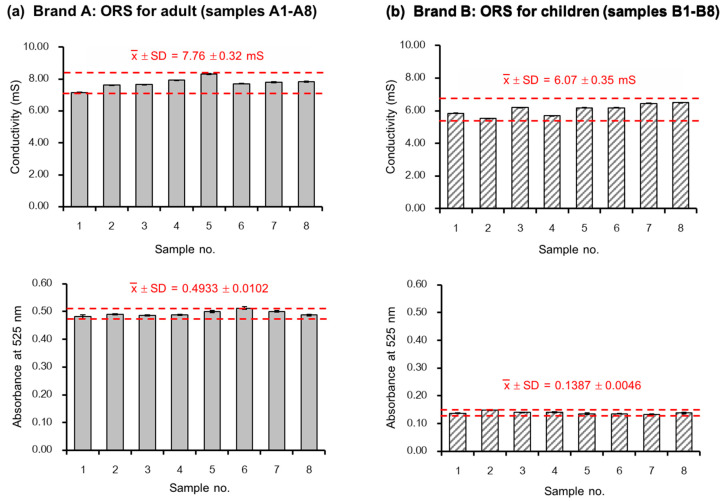
Bar graphs of the mean conductivity (mS cm^−1^) of each sachet (dose) and the mean absorbances of 150.0 mL solutions for Brand A or 120.0 mL solutions of Brand B, with error bars (*n* = 3). The red text shows the means and standard deviations for samples A1–A8 and for samples B1–B8, respectively. The red dotted lines indicate the range for the mean ± 2SD of these two products. (**a**) Eight samples from “Brand A” for adults, labelled net weight of 3.645 g. (**b**) Eight samples from “Brand B” for children, labelled net weight of 3.3 g.

Sixteen sachets of ORS powder products with orange flavoring were employed in the analysis: eight sachets of “Brand A” for adults (A1–A8), and eight sachets of “Brand B” for children under 6 years old (B1–B8). The amount of electrolyte salts in a sachet for children is approximately one half of the amount provided for adults (17% (*w*/*w*) compared to 34% (*w*/*w*)). The powder in each sachet of “Brand A” was totally dissolved in 150.0 mL DI water, whereas the content of “Brand B” was dissolved in 120.0 mL DI water. These volumes were the recommended volumes of water, as given on the sachets. A 100 μL aliquot of each sample was aspirated into the SIA system (see Figure 2). The measured C4D signals were converted into mS cm^−1^, using the calibration line in Figure S1a. The PEDD signals were similarly converted to absorbance units, and indicated the contents of the orange colorants in the ORS powders. The results are shown in Figure 4a,b, respectively.

The bar graphs show the mean values of the C4D measurements, converted to mS cm^−1^ unit, for these two products with values in red text together with their standard deviations. The small variation of the conductivity and the absorbance about their mean values (within ± 2SD) is a measurement of the consistency of the contents of each sachet, ensuring consumer protection.

### 2.4. Determination of Creatinine and Measurement of Conductivity of Urine Samples

The electrical conductivity of urine is due to ionic species in urine [55]. Measurement of urine conductivity is a fast and convenient way to ascertain the hydration statuses of athletes [56] and to evaluate the diuretic effects of medicinal administration [57]. Determination of creatinine concentration in urine is one of the commonly required parameters for medical diagnosis. The amount of creatinine in urine is also used to correct for variation in the volume of spot urine collected for measurements of excreted drugs.

#### 2.4.1. Zone Sequence and Signal Profiles

The SIA system in Figure 2 was employed for the sequential measurements of creatinine and the conductivity of human urine. For this application, port 4 of the selection valve was connected to the reagent reservoir containing the Jaffé reagent of alkaline picrate. This reagent is used for colorimetric detection of creatinine with PEDD. Similarly to the previous application of ORS samples, C4D was used to directly measure the conductivity of urine.

**Figure 5:**
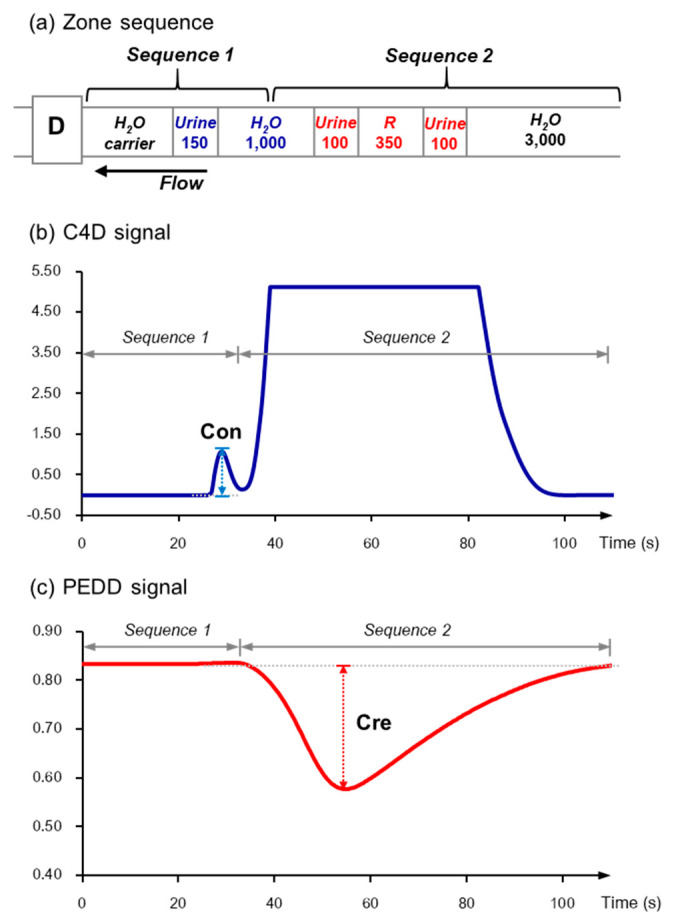
Measurements of urine conductivity and determination of creatinine using SIA equipped with the “2-in-1” C4D-PEDD detector. (**a**) Zone sequence of sample and reagent segments with aspirated volumes in microliters (D: detector). (**b**) Peak profile of urine conductivity (Con) from C4D. The large peak is due to the conductivity of the ions in the mixed zones of Sequence 2. (**c**) Peak profile of creatinine signal (Cre) from PEDD.

Preliminary experiments were carried out (data not shown) to optimize the zone sequence required for sequential detection of the urine conductivity followed by the colorimetric measurement of the complex formed between creatinine and the Jaffé reagent. The final selected zone sequence is shown in Figure 5a. The first sequence, designated Sequence 1, consists of three liquid segments; i.e., H_2_O carrier (in the flow line)|urine (150 µL)|water (1000 µL). Sequence 1 contains the plug of urine which is propelled to the flow cell. Sequence 2, directly following Sequence 1, consists of four liquid segments; i.e., urine (100 µL)|Jaffe reagent R (350 µL)|urine (100 µL)|H_2_O (3000 µL). 

An example of the signal profile recorded from the C4D detector is shown in Figure 5b. The peak labelled “Con” corresponds to the passage of the urine segment (150 µL) of Sequence 1. The large signal following it is the large conductivity of the mixed three segments in “Sequence 2”—viz., urine (100 µL) |R (350 µL) | urine (100 µL), which is not used in the analysis of the C4D data. When all liquid zones comprising Sequence 1 have moved out of the flow cell, the signal (labelled as “Cre”) for the product of the reaction of the two urine plugs with the plug of the Jaffé reagent is seen via the PEDD channel, as shown in Figure 5c. Note that there is no absorbance signal for the urine zone of the leading Sequence 1.

It was necessary to optimize the volume of the water segment (labelled as H_2_O 1000 µL in Sequence 1) separating the first urine sample plug from the urine segments for the creatinine measurements. When using a segment of water less than 1000 µL, the two signal peaks were not baseline separated. For photometric measurements, the schlieren effect may occur at the interface of the sample zone and the water carrier due to differences in the refractive indices [19,44,58,59]. This was also observed with the PEDD signal for some urine samples. For these samples, a small sharp peak was observed in front of the large creatinine peak (see examples in Figure S3 in the Supplementary materials). Since the peak height of the PEDD signal was employed, the schlieren signal did not affect the analysis.

#### 2.4.2. Analysis of Human Urine Samples

Employing the optimized sequence of sample and reagent introduction, standard solutions containing NaCl and creatinine (at concentrations covering the range of values expected for the diluted urine samples) were measured. It should be noted that the calibration equations for the C4D are not the same for the ORS and urine measurements (see Figure S1) due to the different volumes of sample aspirated into the flow-line (100 μL for ORS and 150 μL for urine) leading to different dispersion of the sample plug [60].

For PEDD, the negative of the difference (−ΔV) values of the signal minimum (in volts, V) from the baseline value (“Cre” in Figure 5c) were plotted against the concentrations of standard creatinine. The calibration of PEDD was y = ((2.06 ± 0.01) × 10^−3^)x + ((1.37 ± 0.01) × 10^−1^): *r*^2^ = 0.999). The C4D and PEDD calibration equations were used to calculate urine conductivity and the concentration of creatinine in urine, respectively. The results are shown in Table 1.

The determination of creatinine using the SIA flow-cell was also compared with a batch method employing the Jaffé reaction [61,62,63]. Data in Table 1 show good agreement between the two methods, as confirmed by the statistical paired *t*-test (*t*_stat_ = 2.09, *t*_crit_ = 2.45).

#### 2.4.3. Analytical Features

The analysis is simple with only dilution of urine with water (10-fold or 20-fold). The sample can be directly injected into the SIA system shown in Figure 2 and the data obtained within 2 min for a single injection. Linearity ranges are 0.37–3.93 mS cm^−1^ for the C4D and 5–100 mg L^−1^ for creatinine, respectively. Limits of quantitation (LOQs) for conductivity and creatinine were found to be 0.33 mS cm^−1^ (10 S/N) and 2.38 mg L^−1^ (10 SD intercept/slope), respectively. Compared to other Jaffé methods operated using SIA [64] or cross injection analysis (CIA) [10] flow platforms, this method gave a significantly lower LOQ for creatinine (2.38 mg L^−1^ compared with 11.7 mg L^−1^ and 20 mg L^−1^ for the other SIA and CIA flow platforms, respectively). Ten replicate analyses of a standard mixture of 10 mM NaCl and 10 mg L^−1^ creatinine were carried out to investigate the precision of the method. The flow method gave precisions for conductivity and creatinine of 1.2% RSD and 1.4% RSD, respectively. The precisions in analysis of human urine samples (*n* = 8) were %RSDs of 0.03–0.50% and 0.68–2.6%, for conductivity and creatinine, respectively. The precision for creatinine is improved from previously reported SIA [64] and CIA [10] methods. There are no other flow-based methods for direct analysis of urine conductivity. We therefore were not able to compare our method in the terms of LOQ and precision. Conductivity detection is integrated in flow cytometer (FC) instruments [65,66]. However, there is no information of LOQ and precision for conductivity, since the major role of FC instruments is for cell counting.

## 3. Materials and Methods

### 3.1. Chemicals, Reagents and Samples

All chemicals used in this work were analytical-reagent grade. Deionized (DI) Milli-Q^®^ water was used throughout.

#### 3.1.1. Preparation of Standard Solutions and Oral Rehydration Salt Samples

For the analysis of an oral rehydration salt (ORS) sample, a stock solution of 1.00 M NaCl was prepared using NaCl crystals (Fluka, Switzerland). A stock solution of Sunset Yellow was prepared by diluting a 220 µL aliquot of the liquid food colorant (Winner’s orange color, Greathill Co., Ltd., Bangkok, Thailand) in 100.0 mL of water. The standard working solutions of 5–50 mM NaCl were prepared by adding an appropriate volume of the stock dye solution to obtain the desirable absorbance values of the saline solution (see caption of Figure 3 for the absorbance values). The absorbance was measured on a Lambda 25 UV-Vis spectrophotometer (Perkin Elmer, Waltham, MA, USA) and the conductivity measured using a commercial conductivity probe (Model 145, Thermo Orion, Beverly, MA, USA). These standard solutions were used to construct the calibration graphs for the C4D measurements and the amount of colorant in the ORS product, respectively.

Two brands of local ORS products (“Brand A”: 8 samples, and “Brand B”: 8 samples) produced by two different pharmaceutical companies were purchased and analyzed for the salt contents. “Brand A” products are for adults, whilst “Brand B” is labelled for children. For a single dose, a patient is recommended to empty the contents of the sachet (3.3 g net weight per sachet) and dissolve them in 150 mL drinking water. “Brand B” ORS product for children (3.645 g net weight per sachet) is recommended to be dissolved in 120 mL of drinking water. These samples were dissolved in DI water, as recommended, and injected into the SIA system shown in Figure 2.

#### 3.1.2. Standard Solutions, Reagents and Samples for Urine Analysis

Stock creatinine solution at 5.00 g L^−1^ was prepared by dissolving 500 mg (to the nearest mg) of anhydrous creatinine (Sigma, St. Louis, MO, USA) in 100.0 mL of water. This stock creatinine solution was mixed with appropriate volumes of the stock standard 1.00 M NaCl solution (Section 3.1.1) to obtain a series of standard solutions for calibration; i.e., 2 mM NaCl+5 mg L^−1^ creatinine, 10 mM NaCl+20 mg L^−1^ creatinine, 30 mM NaCl+50 mg L^−1^ creatinine, 35 mM NaCl+75 mg L^−1^ creatinine and 40 mM NaCl+100 mg L^−1^ creatinine, respectively.

The Jaffé reagent was prepared by mixing 9.6 mL of aqueous saturated solution of picric acid (ca. 52 mM) with 4.0 mL of 2.5 M NaOH and 11.4 mL DI water, to give the alkaline picrate reagent solution (20 mM picrate in 0.4 M NaOH). Urine samples were anonymous samples from the National Doping Control Centre (NDCC), Mahidol University, Thailand. Before analysis, urine samples were diluted at least 10-fold with DI water. The absorbance of the creatinine was measured on a Lambda 25 UV–Vis spectrophotometer (Perkin Elmer, Waltham, MA, USA).

### 3.2. Flow Cell with Dual PEDD-C4D Detectors

The flow cell was made by drilling a square acrylic block (2 × 2 × 1.5 cm) to contain a cylindrical flow channel (0.3 cm i.d.) in a Z-configuration. The inlet and the outlet of a flow cell were fitted with screw fittings for inserting and connecting the PEEK tubes (0.75 mm i.d.). Silver conducting ink (SPI Supplies, West Chester, PA, USA) was painted on the exterior of these tubes to make electrodes E1 and E2 of the C4D (Figure 1a). The width of an electrode was 1 cm. The length of tubing between the inner edges of electrodes E1 and E2 was 4.5 cm. The C4D detector is an in-house, purpose-built electronic device. A sinusoidal AC voltage (V_AC_) from the device (XR-2206 monolithic function generator, EXAR Corporation, Fremont, CA, USA) at 500 kHz and 10 V_pp_ was applied to electrode E1. The resulting AC current was measured at the E2 electrode using the pick-up amplifier (OPA655, Burr-Brown, Tucson, AZ, USA), which was connected to a rectifier (AD630, Analog Devices, Norwood, MA, USA) [67]. The final DC output voltage was recorded using an e-corder 210 (eDaq, Denistone East, NSW, Australia). The Z-flow cell and the pre-amplifier were installed inside an aluminum case, as shown in b, for electrical shielding of the C4D detection.

The paired emitter–detector diode (PEDD) detector [45] is integrated into the Z-flow cell for photometric detection. The PEDD detector consists of a pair of green light emitting diodes (InGaN LED, λ_D_ 525 nm, ∅ 0.5 cm, Kingbright, New Taipei City, Taiwan). The vertical channel shown in Figure 1a,b is the light path for the optical detection by PEDD. An acrylic disc (∅ 0.5 cm and 0.1 cm thick) is glued at each end of the channel as optical windows and to seal the channel. One of the LEDs is used as the light source, whilst the second LED, placed at the opposite end of the light path, is the light detector. The optical path length of this cell is 1 cm. The LED light source is connected in series to a 100 ohm current limiting resistor and a DC voltage power supply (Model BK-1502D+, Baku, China), set at 8.00 V. A 6-digit multimeter (10 MΩ impedance, Model 8845A, Fluke, Everett, WA, USA) is used to directly measure the output signal from the LED detector to a computer via a RS232 to USB cable, since the input impedance of the e-recorder was too low. LabVIEW 8.0^TM^ software is employed for recording and displaying the PEDD signal on a notebook computer. 

### 3.3. Analysis by SIA with Dual-Detection Flow Cell

The SIA system in Figure 2 was employed for all work. A syringe pump (PSD/4 Hamilton, Reno, NV, USA) with an 8-port selection valve (MVP Hamilton, Reno, NV, USA) was used to control the carrier and reagent flows. Teflon tubes (0.1 cm i.d.) were used as flow lines. The schematics of the C4D and PEDD detectors are depicted in Figure 2. The inlet of flow-through detection cell (Figure 2) was connected to the SIA system via port 3 of the selection valve (SV) using 20-cm PTFE tubing. The MGC-MPV LMPro (version 5.2) software was used for controlling the syringe pump and the selection valve of the SIA system. The flow protocols for the analysis of ORS samples and for urine samples are given in Tables S1 and S2, respectively.

## 4. Conclusions

We have constructed a flow cell that incorporates a PEDD and C4D as dual detectors that can measure the absorbance and conductivity of a solution simultaneously. This was demonstrated by the measurements of the conductivity and absorbance (at 525 nm) of a solution of dissolved oral rehydration salts (ORS). Eight samples from two commercial brands were analyzed. The results show that there were no statistical differences between the measured conductivities and absorbances of the samples intra-brand. This is a useful method for consumer protection of ORS products.

When the dual-detector is linked to a SIA system, the conductivity of the sample can be directly measured in one flow sequence, whilst the ensuing flow sequence contains the sample that has reacted with a color-forming reagent, and the absorbance is measured in a sequential manner. Thus, two variables of a sample can be measured, such as conductivity of urine and its creatinine content after reacting with the Jaffé reagent added on-line. The conductivity values are useful for monitoring the hydration status of an athlete. Urine creatinine content is an index of various health problems and is employed as the normalization factor in the measurement of excreted drugs in spot urine. The creatinine concentrations were validated against a batch method using the Jaffé reagent and a spectrophotometer.

## Figures and Tables

**Table 1:** Analysis of conductivity and creatinine of human urine samples.

Sample	Conductivity (mS cm^−1^)	Creatinine (mg L^−1^) *	
	C4D Detector (*n* = 3)	PEDD Detector (*n* = 3)	Spectrophometer (*n* = 3)
S1	19.18 ± 0.03	(1.06 ± 0.02) × 10^3^	(1.08 ± 0.01) × 10^3^
S2	27.10 ± 0.12	(1.40 ± 0.02) × 10^3^	(1.41 ± 0.03) × 10^3^
S3	26.57 ± 0.05	(1.54 ± 0.01) × 10^3^	(1.56 ± 0.02) × 10^3^
S4	31.60 ± 0.01	(1.17 ± 0.02) × 10^3^	(1.14 ± 0.02) × 10^3^
S5	12.42 ± 0.02	(7.36 ± 0.05) × 10^2^	(7.13 ± 0.07) × 10^2^
S6	10.07 ± 0.02	(5.60 ± 0.05) × 10^2^	(5.06 ± 0.05) × 10^2^
S7	4.99 ± 0.02	(1.52 ± 0.04) × 10^2^	(1.22 ± 0.01) × 10^2^
S8	11.96 ± 0.06	(4.39 ± 0.06) × 10^2^	(4.09 ± 0.06) × 10^2^

* Paired *t*-test: *t*_stat_ = 2.09, *t*_crit_ = 2.45.

## Supplementary Materials

The following are available online. Figure S1: (**a**) The calibration plot of C4D signal (V) against conductivity (mS cm^−1^) for the ORS analysis. The equation of the regression line is y(V) = (0.47 ± 0.01)x − (0.27 ± 0.02), *r*^2^ = 0.9993. (**b**) The calibration plot of C4D signal (V) against conductivity (mS cm^−1^) for urine measurement. The equation of the regression line is y(V) = (1.47 ± 0.02)x − (0.47 ± 0.05), *r*^2^ = 0.9996. It should be noted that the two calibration equations are not the same due to the different volumes of sample aspirated into the flow-line (100 μL for ORS and 150 μL for urine) leading to different dispersions of the sample plug. Figure S2: The slopes and intercepts of the calibration lines from the simultaneous C4D and PEDD measurements of a series of saline solutions containing orange dye. Column “a” is from the consecutive measurements using increasing concentrations of the calibration solutions. Column “b” is from the consecutive measurements using decreasing concentrations of the calibration solutions. Figure S3: Examples of triplicate PEDD signals showing small reproducible schlieren peaks appearing before the creatinine peaks of three urine samples. Table S1: Procedure of the SIA system for the analysis of ORS samples. Table S2: Procedure of the SIA system for the analysis of urine samples.
