# Supplementary material for: Dual-Purpose Photometric-Conductivity Detector for Simultaneous and Sequential Measurements in Flow Analysis
Source: Molecules. 2020 May 13;25(10):2284. doi: 10.3390/molecules25102284 (PMC7287826; doi:10.3390/molecules25102284)
Supplement: Supplementary file 1 [file molecules-25-02284-s001.pdf]

Supplementary materials

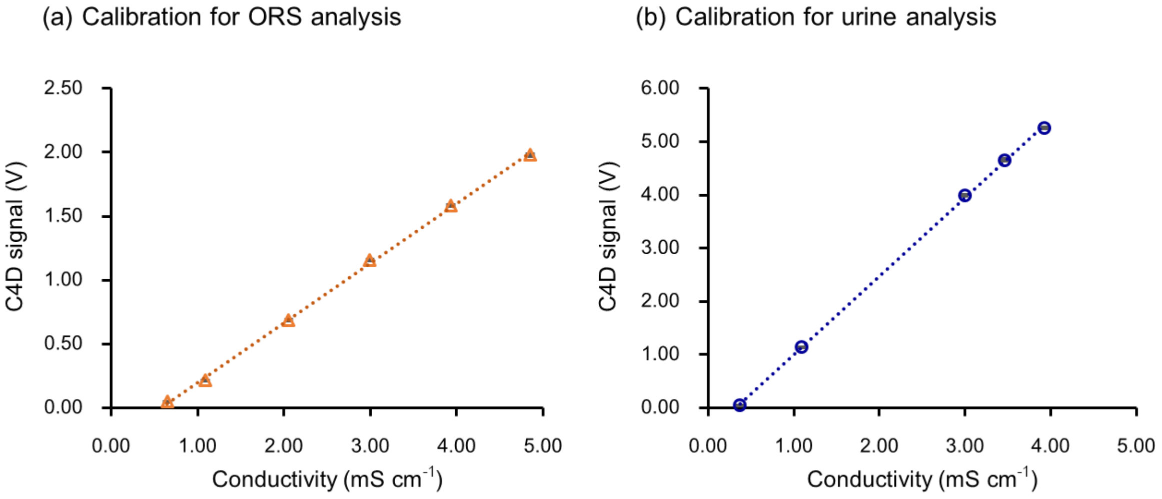

Figure S1. (**a**) The calibration plot of C4D signal (V) against conductivity (mS cm^{-1}) for the ORS analysis. The equation of the regression line is y(V) = y (0.47 ± 0.01)x − (0.27 ± 0.02), r^2 = 0.9993. (**b**) The calibration plot of C4D signal (V) against conductivity (mS cm^{-1}) for urine measurement. The equation of the regression line is y(V) = (1.47 ± 0.02)x − (0.47 ± 0.05), r^2 = 0.9996. It should be noted that the two calibration equations are not the same due to the different volume of sample aspirated into the flow-line (100 µL for ORS and 150 µL for urine) leading to different dispersion of the sample plug.

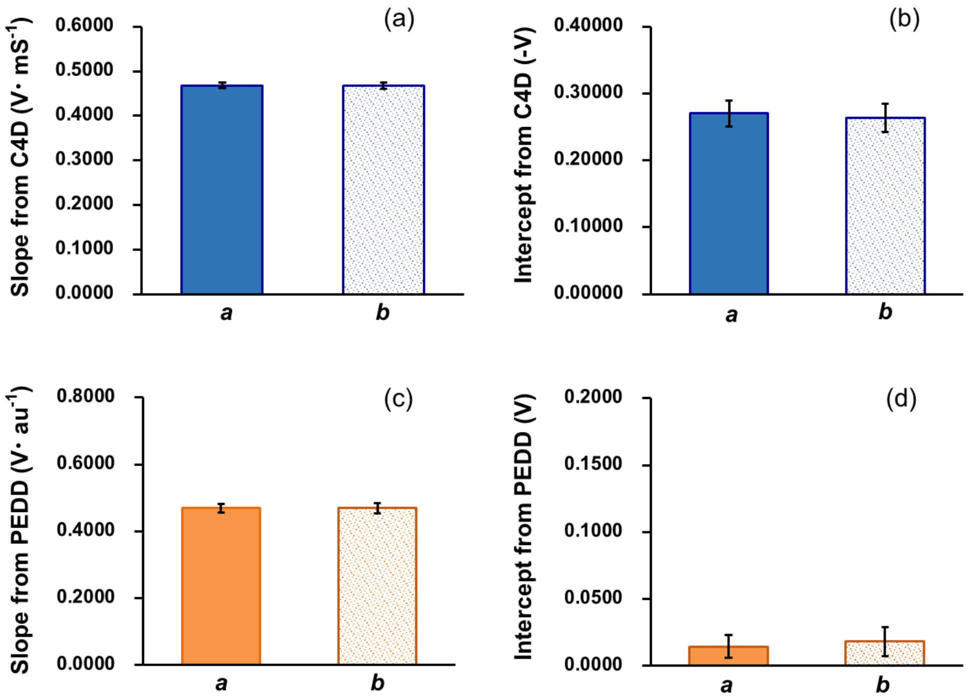

Figure S2. The slopes and intercepts of the calibration lines from the simultaneous C4D and PEDD measurements of a series of saline solutions containing orange dye. Column "a" is from the consecutive measurements using increasing concentration of the calibration solutions. Column "b" is from the consecutive measurements using decreasing concentration of the calibration solution.

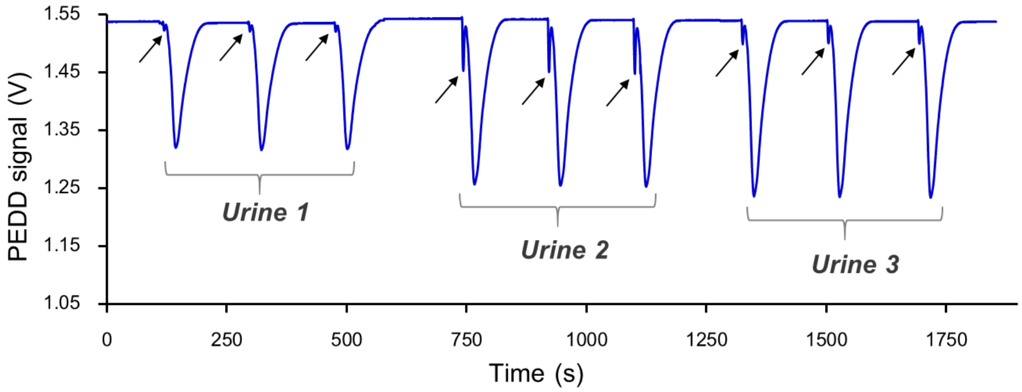

Figure S3. Examples of triplicate PEDD signals showing small reproducible schlieren peaks appearing before the creatinine peak of three urine samples.

Table S1. Procedure of the SIA system for the analysis of ORS samples.

Step	Syringe Valve Port	Selection Valve Port	Flow Rate (μL s^{-1})	Volume (μL)	Description
1	In	-	100	1000	Fill syringe with DI H_2O.
2	Out	5	50	100	Aspirate std./ORS sample solution.
3	Out	3	50	1100	Propel the liquid plugs to flow cell for simultaneous monitoring of conductivity using C4D and absorbance using PEDD (λ 525 nm).

Table S2. Procedure of the SIA system for the analysis of urine samples.

Step	Syringe Valve Port	Selection Valve Port	Flow Rate (μL s^{-1})	Volume (μL)	Description
1	In	-	100	3000	Fill syringe with DI H_2O.
2	Out	5	50	100	Aspirate plug 1 of std./urine sample solution.
3	Out	4	50	350	Aspirate alkaline picrate reagent.
4	Out	5	50	100	Aspirate plug 2 of std./urine sample solution.
5	Out	8	100	1000	Aspirate DI water.
6	Out	5	50	150	Aspirate plug 3 of std./urine sample.
7	Out	3	50	4700	Dispense all liquid plugs to flow cell for sequential monitoring of conductivity using C4D and creatinine complex using PEDD (λ 525 nm).
